# Effect of Deforestation and Land Use Changes on Mosquito Productivity and Development in Western Kenya Highlands: Implication for Malaria Risk

**DOI:** 10.3389/fpubh.2016.00238

**Published:** 2016-10-26

**Authors:** Eliningaya J. Kweka, Epiphania E. Kimaro, Stephen Munga

**Affiliations:** ^1^Mosquito Section, Division of Livestock and Human Diseases Vector Control, Tropical Pesticides Research Institute, Arusha, Tanzania; ^2^Department of Medical Parasitology and Entomology, Catholic University of Health and Allied Sciences, Mwanza, Tanzania; ^3^Centre for Global Health Research, Kenya Medical Research Institute, Kisumu, Kenya

**Keywords:** highland, *Anopheles gambiae* ssp., habitat productivity, land use, topography, temperature, gonotrophic cycle

## Abstract

**Background:**

African highlands were known to be free of malaria for the past 50 years. However, the ever growing human population in the highlands of Africa has led to the deforestation and land coverage changes to create space for more land for cultivation, grazing, and house construction materials needs. This has lead to the creation of suitable breeding habitats, which are in open places. Decrease of canopy and forest cover has led to increased temperature both in outdoors and indoors in deforested areas. This increased temperature has resulted in the shortening of developmental stages of aquatic stages of mosquitoes and sporogony development in adult mosquitoes.

**Method:**

Assessment of the effects of deforestation and land coverage changes (decrease), which leads to temperature changes and subsequently increases survivorship of adults and sporogony development in adult mosquitoes’ body was gathered from previous data collected from 2003 to 2012 using different analysis techniques. Habitats productivity, species dynamics and abundance, mosquitoes feeding rates, and sporogony development are presented in relation to temperature changes.

**Results:**

The effects of temperature rise due to land cover changes in highlands of western Kenya on larval developmental rates, adult sporogony developments, and malaria risk in human population were derived. Vector species dynamics and abundance in relation to land use changes have been found to change with time.

**Conclusion:**

This study found that, land cover changes is a key driver for the temperature rise in African highlands and increases the rate of malaria vectors *Anopheles gambiae* ssp., *An. Funestus*, and *An. arabiensis* colonizing the highlands. It has also significantly enhanced sporogony development rate and adult vector survival and therefore the risk of malaria transmission in the highlands.

## Introduction

In the recent past, the African highland sites were known to be malaria-free zones. Only sporadic malaria outbreaks were recorded earlier between 1920 and 1950 in the East African highlands (1, 2). However, since 1988, malaria epidemics occurred nearly every year at different sites of African highlands and are a great concern of public health (3–7). These frequent outbreaks have raised concerns that highland malaria has been on the rise (8–12) in the recent past.

The highlands have come under intense pressure from increased population growth. This has increased the demand resulting in tremendous changes in land use practices (13–15). In western Kenya highlands, forests have been cleared to create farmlands, pasture, and homesteads, while swamps have been cultivated to increase agricultural production. These environmental degradation processes, which mainly occur as a result of anthropogenic activities, change the ecological balance and context thus providing suitable habitats for disease vectors to breed, develop, and transmit disease.

Each species occupies a particular ecological niche, and vector species subpopulations are distinct behaviorally and genetically as they adapt to man-made environments (16, 17). However, the major causes of spatial differences in vector distribution and abundance in such heterogeneous environments has not been investigated in the highlands. In lowland areas around Lake Victoria in western Kenya, Minakawa and others detected significant spatial heterogeneity in species composition of anopheline larvae composition in Suba district (18). However, they did not identify environmental variables that determine the occurrence and relative abundance of anopheline larvae.

Several postulates have been put forward to explain increase in malaria epidemics in high altitude areas: land use changes (4, 9, 10), changes in climate (4, 19), and demographic patterns (5, 17). Land cover changes have been suggested to cause increased risks of malaria transmission in high altitude areas in several parts of the world (9, 10, 12, 20). Hoof prints in newly cleared areas form puddles during rains and are conducive for breeding of *Anopheles gambiae* (18). Replacement of natural swamp vegetation with agricultural crops also lead to increased temperatures, which was suggested to have been responsible for elevated malaria transmission risk in cultivated areas in high altitude areas in Uganda, central Africa, and Madagascar (5, 9). The destruction of tropical forests in west Africa lead to increased risks of malaria transmission and other diseases (17).

Although there is great diversity of anopheline species in the highland areas of Africa members of the *An. gambiae* complex and *Anopheles funestus* are the principal vectors of malaria, as is true for most of the continent (21–24). This study explored the effect of deforestation and land cover changes on effect of mosquitoes’ life cycle and infectivity influences.

## Materials and Methods

### Study Site Location

**Figure 1:**
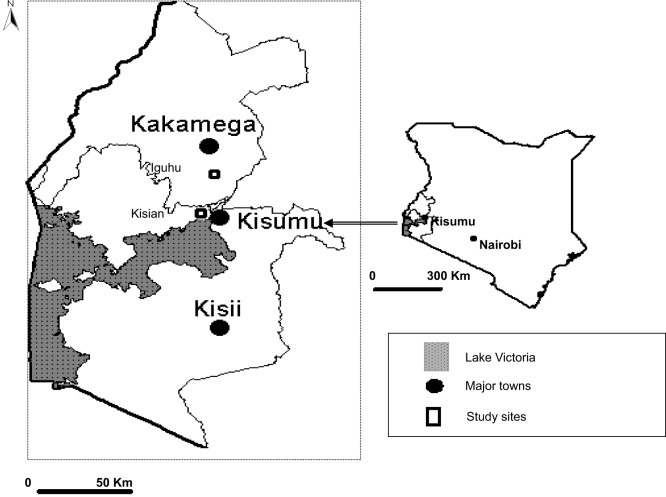
**A map showing the selected sites in western Kenya highlands**.

The study area is situated at Iguhu, a highland area in Kakamega and Vihiga districts of Western Kenya Province (Figure 1). The study sites were located within a grid of 4 × 4 km^2^ and at 0°17′N, 34°74′E and densely populated with approximately 3000 inhabitants. The area has an average population growth rate of 2.98% per annum. Iguhu was selected as a highland area based on previous work that had been done in the area during 1997/1998 El Nino episode in Kenya (25). Like many highland areas in western Kenya, increase in human settlements has led to rapid changes on land use and land cover. These changes in land use practices continue to create more space to meet other human needs such as pasture fields and farmland.

### Estimation of Relative Habitat Productivity (Emerging Adults)

**Figure 2:**
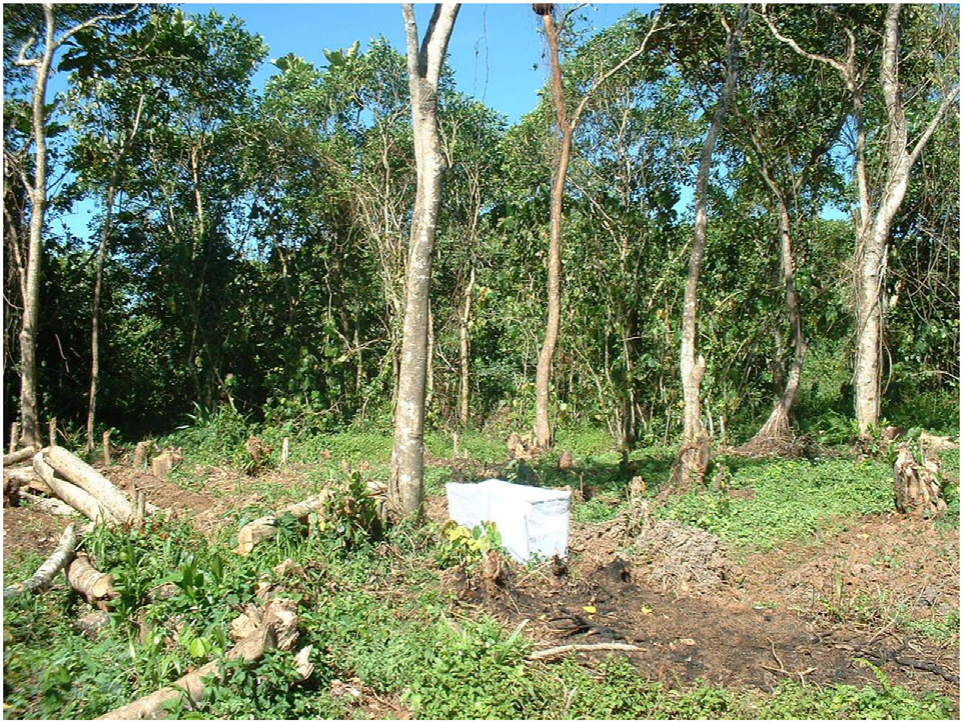
**Remnants of indigenous forest undergoing deforestation, in the foreground is a drainage ditch with an emergence trap for estimating relative habitat productivity**.

Relative larval habitat productivity in terms of emerging adult mosquitoes was estimated monthly in different land cover types (cultivated and natural swamps, and forest) using collapsible emergence traps (26, 27) monthly from July 2002 to July 2003 and other part on 2008–2010. The traps had a surface area of 1 and 0.5 m^2^ (Figure 2). The traps were placed over 30 randomly selected habitats (10 habitats in each land cover type) for a period of 7 days while being moved daily within the habitat to maintain dynamics of the habitats. Emerging adults were collected the following day by aspirators, preserved, and identified using morphological keys. Members of the *An. gambiae* complex were identified by rDNA–PCR (28). Productivity of each land use type was calculated as number of emerging adults per square meter per week. The following habitats parameters were also measured: depth, width, size, canopy cover, algae, grass, and debris cover. Surface area of the larval habitat was calculated as a function of width and length. Canopy cover was measured by taking four readings at right angles at each habitat using a densitometer. Water temperature was recorded using Stowaway tidbit data logger (Onset Computers, MA, USA) at hourly interval.

### Indoor Microclimate Changes Induced by Land Use and Land Cover

Microclimate is defined as the climate of a very small or restricted area, especially when this differs from the climate of the surrounding area. Houses were selected in the forest and deforested areas of Iguhu and in the lowland site of Kisian, Kisumu. Houses selected in both the highland and lowland areas were those that had corrugated iron roofing. Four houses were selected at each of the three sites for the study. The minimum distance between houses in the forest and deforested areas was about 2.5 km. Houses within the same area (forest, deforested, or the lowland sites) were selected to be over 300 m apart to allow the capture of the microclimate of houses of a larger area within the study sites. Microclimate data were collected using HOBO^®^ data loggers placed inside and outside the houses in Stevenson’s screens all placed 2 m from the ground.

### Effects of Land Use and Land Cover on Gonotrophic Cycle Duration

Mosquitoes used in this experiment were *An. gambiae* from Iguhu, western Kenya. Standard cages of sizes 30 cm × 30 cm × 30 cm containing hundred 4-day-old virgin females of *An. gambiae* were hanged inside the house. Each site had three houses selected, and inside each a cage was hung. The mosquitoes were fed on human blood for 30 min (a volunteer). Unfed mosquitoes were taken out of the cage. Similar numbers of *An. gambiae* ssp. males from insectary were introduced in the cage with fully fed females for mating purposes. In 24-h time, each blood-fed and mated female was transferred into a cup for oviposition. Each female was provided with oviposition substrates and placed on the table inside a sleeping room. Each paper cup was covered with polystyrene material. Sugar solution was dispensed using wet cotton placed on top of each cup. The eggs laid in each gonotrophic cycle were counted. Successfully oviposited mosquitoes were given a second blood meal. Microclimate data were collected using HOBO^®^ data loggers placed in the bedrooms of the houses and outside the houses in Stevenson’s screens all placed 2 m from the ground.

### Deforestation Effects on the Survivorship and Reproductive Fitness of *Anopheles gambiae*

Three houses each in the forest and deforested area were used for this study during the dry season, and four houses each were used during the rainy season in all the forest, deforest, and lowland area. Also, 100 newly emerged female and 100 male *An. gambiae* raised in the insectary in Kisian were placed in a 30 cm × 30 cm × 30 cm metal-framed cage covered with polystyrene netting. Cages were suspended from the roof, 2 m above the ground, in the bedrooms of the selected houses. Human blood from the hand was provided to the mosquitoes for about 15 min every 2 days in the mornings. An oviposition substrate consisting of a petri dish lined with a Whatman filter paper was provided. Sugar solution 10% was dispensed using cotton wool in each cage. Number of eggs laid was counted daily. All dead mosquitoes were taken off the cages. Each cage had a HOBO^®^ data logger to record indoor temperature and humidity.

### Data Analysis

Effect of different land use types on larvae abundance was done using one-way analysis of variance (ANOVA) for comparisons of larval abundances in different land use, cropping season, vegetation cover, seasonality, cropping season, and breeding habitat type. Where significant differences were observed, the means were separated by Tukey–HSD test.

Estimation of relative habitat productivity (emerging adults) was analyzed using stepwise multiple logistic regressions. The effect of land use and seasons on the habitat productivity was analyzed by two-way ANOVA. Comparison of the habitats’ productivity between wet and dry seasons was computed by Student’s *t*-test. Effects of land use and land cover on gonotrophic cycle duration of female mosquitoes and indoor microclimate changes induced by land use and land cover was done using land use cover. The number of eggs laid in each type of land cover and the proportion of mosquitoes that laid eggs was compared using the Chi-square (χ^2^) test. Non-parametric Kruskal–Wallis rank sum tests were used to determine the effects of land cover (forest vs. deforested) on the mean duration of the gonotrophic cycle of *An. gambiae*. The analysis of the effect of land cover types on fecundity in different season was done by *t*-test.

Analysis of variance with repeated measure was used to assess the effects of deforestation on the survivorship and reproductive fitness of *An. gambiae* in western Kenya highland on outdoor and indoor temperature and relative humidity. The age-dependent mortality comparison was calculated using *t*-test between forest and deforested and between seasons. The difference was considered significant when the *p*-value was below 0.05.

### Ethics Statement

The Kenya Medical Research Institute, National Ethical Review Committee, and University of California, Irvine ethical review board, approved the ethical clearance for this study under the main project “Ecology of African highland malaria (II), with SSC No. 1382.”

## Results

### Effect of Different Land Use Types, Seasonality, and Agriculture on Larval Abundance

#### Vegetation Cover and Larval Abundance

The abundance of plants vegetation cover in habitats influenced significantly *An. gambiae* s.l. larvae abundance (*F*_15.18, 2_, *p* ≤ 0.001), and other anopheline species and other anopheles species (*F*_3.40, 2_, *p* = 0.034). Mosquito larval abundance associate negatively with increased habitat vegetations, Culicine species (*F*_1.58, 2_, *p* = 0.207), and *An. funestus* (*F*_2.69, 2_, *p* = 0.069). In grouping all larvae species together, the increase in grass cover associated highly with the decrease of larvae abundance (*F*_15.25, 2_, *p* ≤ 0.001).

#### Cropping Cycles and Larvae Abundance

In all three crop cycle (land preparation, crops weeding, and plant flowering) *An. gambiae* s.l. (*F*_8.14, 2_, *p* ≤ 0.001), other anopheline (*F*_12.6, 2_, *p* ≤ 0.001) and Culicines (*F*_21.1, 2_, *p* ≤ 0.001) larvae abundance were significantly different between cropping cycle, while *An. funestus* (*F*_30.2, 2_, *p* = 0.277) were not significantly different.

#### Seasonality and Larvae Abundance

In different seasons (rainy, short rainy, and dry seasons) have found that, *An. gambiae* s.l. had no significant difference (*F*_2.14, 2_, *p* = 0.119), while other anopheline (*F*_13.6, 2_, *p* = 0.003), Culicines (*F*_18.7, 2_, *p* = 0.007), and *An. funestus* (*F*_6.38, 2_, *p* = 0.046) were statistically different among seasons.

#### Habitats Types and Larval Abundance

In all habitat types, the larvae abundance was significantly higher in *An. gambiae* s.l. (*F* = 2.80, DF = 3, *p* = 0.004), *An. funestus* (*p* ≤ 0.001), other anopheline (*p* ≤ 0.001), and *Culicine* (*p* ≤ 0.001) among the habitats.

#### Land Use Types and Larval Abundance

Pasture had statistically significant higher abundance of *An. gambiae* s.l. (*F*_4.23, 1_, *p* = 0.040), other anophelines (*F*_13.44, 1_, *p* ≤ 0.001), and Culicines species (*F*_4.52, 1_, *p* = 0.034) than in farmland, while *An. funestus* (*F*_0.83, 1_, *p* = 0.363) had no statistically significant differences in larvae abundance between the land use types. In grouping all larvae species together, pasture land use type had significantly higher larval abundance than farmland (*F*_10.05, 1_, *p* = 0.002).

#### Estimation of Relative Habitat Productivity (Emerging Adults)

The principal malaria vector *An. gambiae* ssp. were exclusively found to breed in cultivated swamps with the other species of *Anopheles squamosus, Anopheles coustani, Anopheles Implexus*, and *Anopheles maculipalpis* being recovered from emergence traps both in the forested and natural swamp habitats. Final model stepwise multiple logistic regression of the productivity and the environmental variables measured did not explain much of the productivity of *An. gambiae*. Variables such as debris cover land use, which is associated with canopy cover and season, were found to explain in small proportion productivity of this species.

### Effects of Land Use and Cover on Gonotrophic Cycle Duration of Female Mosquitoes

#### Impact of Deforestation on Relative Humidity, Outdoors, and Indoor Temperature

It was found that, temperature outdoor was 0.4°C higher during dry and rainy seasons in deforested area than in forested area. The indoor average temperature was 1.2°C higher in deforested area than forested areas during dry season (*F*_151.04, 1_, *p* < 0.001), while the outdoor temperature variations between forested and deforested area was 0.7°C higher during the rainy season (*F*_20.26, 1_, *p* < 0.001). The average outdoor temperature in the lowland area was 3.1°C higher than the highland deforested area during the rainy season (*F*_225.34, 1_, *p* < 0.001), while the average indoor temperature was 2.8°C higher (*F*_252.95, 1_, *p* < 0.001). Land cover types also affected indoor relative humidity in the highland.

#### Deforestation Effects on Mosquito Survivorship

The life span of mosquitoes was found to be longer in forested areas than those in deforested areas of western Kenya highlands. During the rainy season, the survival rates were higher in forested area, than in deforested area (χ^2^ = 73.54, *p* < 0.001), similar scenario was observed during the dry season (χ^2^ = 13.11, *p* < 0.001). The survival period in deforested lowlands area was shorter than in deforested area of the highlands (χ^2^ = 27.16, *p* < 0.001).

### Effects of Microclimatic Changes Due to Deforestation on the Survivorship and Reproductive Fitness of *Anopheles gambiae*

#### Effects of Deforestation on Gonotrophic Cycle Duration

During the dry season, mosquitoes (42.7%) in the forest area laid significantly fewer eggs than those in the deforested area (χ^2^ = 7.27, DF = 1, *p* < 0.01). The average duration of the first gonotrophic cycle in the forest area was 4.6 days post blood feeding, 1.7 days (59%) longer than those in the deforested area (2.9 days) (*Z* = 7.72, *p* < 0.001). The average duration of the second gonotrophic cycle in the forest area was 3.0 days post second blood meal, 0.9 days (43%) longer than those in the deforested area (*Z* = 3.62, *p* < 0.001). The number of eggs laid during first gonotrophic cycle in the rainy season was not statistically different between forested and deforested areas (χ^2^ = 1.97, *p* > 0.05). During all the seasons, the gonotrophic cycle was longer in forested area than in deforested area (*Z* = 3.24, *p* < 0.001).

## Discussion

Understanding the effects of land use, and land cover changes, and succession is important in designing an effective targeted malaria control program. This helps in knowing if a larval habitat is most productive and when it should be targeted for maximum reduction of adult population. Currently, there is growing interest of investing on mosquito larval control and the feasibility of reducing malaria vector populations through environmental management, which has been under investigation in different ecological settings in malaria endemic countries (29–34).

Four larval habitat types in two land use types were studied to understand the variation in larval abundance with relation to crop cycle and land use management. This study has demonstrated dominance of *An. gambiae* s.l. over other species for 85 weeks of the larval habitats follow-up in both land use and habitat types. The Simpson diversity index model revealed species homogeneity and heterogeneity in different weeks within the study period. In land use type, pasture land use had higher number *An. gambiae* s.l. larvae abundance than found in farmland as habitats are exposed to sunlight most. These findings have shown similar outcomes as previous studies did in different ecology (13, 24, 35–40). Surveyed larval habitats found that, habitats colonization are species dependant where as *An. funestus* and *Culicines* spp. colonized swamps and large open disused goldmines which has been reported by previous studies (13, 24, 35, 40–43). *An. gambiae* s.l. larvae abundance dominated in hoof print, in small open disused goldmines, and in swamps (13, 24, 35, 40–43).

In farmland, the different cropping cycles’ habitats were dominated by *An. gambiae* s.l. larvae abundance and reduced toward plant flowering season, which is contrary to what other studies found in farmlands that plants’ flowering was the cycle associated with the abundance of *An. gambiae* s.l. in the habitats (44–47). The findings of this study suggest that, the food sources in larval habitats in farmlands are more complex and larvae might have other source of food apart of flowering maize pollens. Hence, the prediction of the food sources for mosquito larvae in this study could not be ascertained by cropping cycles alone. In nature, larvae habitats have been found to have varieties of food sources to support different mosquito species larvae at a point of time (44–47). In different agro-ecosystem it was found that nitrogenous fertilizers’ applications lowers the turbidity of water, which is correlated and proved to associate with the abundance of mosquito larvae (48–52). Studies by McCrae found that, the *An. gambiae* s.l. gravid females prefer to oviposit on turbid water rather than on clear water (53). The lower larval density in early short rainy and in long rainy seasons might be attributed and explained with unexpected flush effect (washing of eggs and larvae from habitats), which was similar with the other studies in western Kenya (24, 54).

The Knowledge of estimation of relative larval habitat productivity and survivorship of *An. gambiae* would be important in planning and implementing larval control measures in high altitude areas in western Kenya. The current study has demonstrated the effect of land cover on larval habitat productivity (emerging adults) and survivorship at a highland area in western Kenya. We have demonstrated that land cover affects the productivity of and larval survivorship of *Anopheles gambaie*. These results confirm the hypothesis that land cover changes in western Kenya highlands alter larval breeding habitat conditions and increases larval survivorship thus accelerating the development rate. Reducing canopy cover may significantly increase productivity of larval breeding habitats in two ways: one is by increasing the availability of open and sunlit pools, which are preferred by *An. gambiae* and two by accelerating the development rate and increasing the survivorship. This may be critical for malaria transmission and may partly explain the upsurge in outbreaks of malaria in the high altitude areas in western Kenya. There were seasonal variations in productivity of *An. Gambiae*, which were likely to have been caused by the amount of rainfall. Higher productivity was recorded during months of high rainfall. These findings are similar to previous studies (55) in rainfall and coincided with abundance of adult mosquitoes. However during the period between September and December 2003, an increase in rainfall did not show a corresponding increase in productivity of *An. gambiae* in the cultivated swamps. During this period, grass cover in these drainage ditches in the cultivated swamps had increased, and habitats may have become unsuitable for breeding of *An. gambiae* even though rainfall levels seemed high to sustain production. At the same time, the dry season marked the end of growing season in the cultivated swamps when the drainage ditches are unattended to by the farmers. Flush effect of rains is also likely to have washed away the larvae. An increase in productivity for the period beginning February to July 2003 may be attributed to clearing of the drainage ditches in the cultivated swamps in preparation for the growing season by the local inhabitants. This may have created suitable breeding habitats for *An. gambiae*. Results of stepwise multiple regression indicated that canopy cover, maximum and minimum temperature, and algae cover affect the productivity of this malaria vector in western Kenya highland. Canopy cover however is highly linked to land cover as forests have higher canopy cover, making the environmental inimical to breeding of this species. Habitats in the open areas receive a lot of sunshine and are likely to have abundant larval food resources such as bacteria and algae (56). Unlike habitats in the open areas, habitats in forest and natural swamp had an average canopy cover of up to 92–97% as estimated by densitometer and visual estimates, respectively. In such shaded habitats, algal growth is likely to be minimal (57). Thus growth and survivorship of *An. gambiae* larvae is likely to be retarded. In the current study, higher productivity of *An. gambiae* was associated with algae (23, 24). Our results are consistent with previous findings in lowland and highland areas in western Kenya in which occurrence of *An. gambiae* and *Anopheles arabiensis* were associated with small, temporary habitats with algae and little aquatic vegetation (36, 42). In the natural conditions, these results of larval habitat productivity may have implications for malaria transmission in western Kenya highlands. Maize is the staple food grown in cultivated swamps, *An. gambiae* larvae may feed on pollen grains and thus emerge as bigger adults with higher vectorial capacity and increase the risk of malaria transmission to a community with low immunity. Previous observations in Ethiopia (58) have demonstrated that larvae of *An. arabiensis*, which fed on pollen grains emerged as larger adults and are capable of imbibing more blood, properties that would be crucial for malaria transmission in the high altitude areas. Also, this study has revealed that, there is an increase in *An. arabiensis* to 33.7% from formerly reported 3.5% in *An. gambiae* sibling species (24), which was implicated by the land use changes and deforestation.

Our results on survivorship of *An. gambiae* larvae under different land cover types in western Kenya must be the first to do so. In the current study, larvae of this malaria vector survived much better in the open areas compared to other land cover type. Density and land cover significantly affected survivorship of mosquito larvae. Larvae in the forested and natural swamp habitats took longer to develop into pupae in the artificial habitats in which larvae were provided with food. In their study, Gimnig et al. (59) suggested that implications for extending development time may include larvae being stranded as the habitat dries up. This suggests that the changes in land cover types in the area may increase the development time and size of mosquitoes. The current study has clearly demonstrated that land cover and density can impact the survivorship and growth of *An. gambiae* under artificial conditions. This may be critical for malaria transmission in this area of unstable malaria. It has been suggested that though density-dependent processes can regulate populations of *An. gambiae* under artificial conditions, it is not known how important these factors can be in natural conditions (59).

The effects of land use and land cover on gonotrophic cycle duration of female mosquitoes demonstrated that, the average ambient temperature in the deforested area in western Kenya highlands was about 0.5°C higher than the forest area over a 10-month period. The average indoor temperature of houses located in the deforested area was 1.8 and 1.2°C higher than those inside the forest area during the dry and rainy seasons, respectively. The relative humidity in the deforested area fluctuated substantially, while the humidity in the forest area was more stable. As a consequence of the indoor temperature increase, the average duration of the first gonotrophic cycle of *An. gambiae* was shorter in both seasons for deforested than forested.

There was significant association between gonotrophic cycle and indoor temperature that could make clear that, temperature is the driving force for the gonotrophic time. The findings suggest that, an increase of temperature by 1°C would decrease the gonotrophic cycle of *An. gambiae* indoors. Similar results have been found by Stern and others that, when temperature increases in highlands of Kericho, more malaria incidences were expected as the effect of better mosquito developmental; conditions including gonotrophic cycle (60).

The results of this study are consistent with those of earlier studies using the mark–release–recapture technique. Rodriguez and others carried out studies on the pacific coast of Mexico and found the gonotrophic cycle of *An. albimanus* to be 4 days for nulliparous mosquitoes and 2 days for parous mosquitoes (61), while Charlwood et al. reported durations of 2.4–3.2 days for *An. koliensis*, 2.7–3.7 for *An. punctulatus*, and 2.1–3.0 days for *An. farauti* in Papua New Guinea (62). The capture–mark–release–recapture technique is unsuitable for study of the gonotrophic cycle duration in the highlands due to low vector densities, which would result in extremely low recapture rates.

The daily man-biting rates affect the two indices used to measure malaria transmission: that is, the vectorial capacity and the entomological inoculation rate (EIR) (63). The vectorial capacity varies as a square of the daily man-biting rates, and EIR varies linearly with the daily man-biting rates. Simple calculations indicated that during the dry season, deforestation caused a net change of the daily man-biting rate from 0.22 to 0.33, which represents a 50% increase in transmission as would be indicated by EIR. However, during the rainy season, the effects of deforestation are lower as the daily biting rate increased from 0.1 to 0.12, representing a 20% increase in transmission assuming all other transmission variables remain the same in the two areas. During the main transmission period, as a result of longer durations of the gonotrophic cycle, the average daily man-biting rate in the highlands was 44% lower compared to the lowlands.

The effect of deforestation on the microclimatic have affected the survivorship and reproductive fitness of *An. gambiae*, it was demonstrated that in the increase in malaria vector reproductive rate was associated with increased temperature due to deforestation and land clearance. Changes in both microclimatic conditions and vector biology depend on land cover types and vary between seasons. Blood meal digestion rate have been found directly proportional to the temperature (64). In addition, lower humidity in houses located in the deforested area may have negatively affected mosquito survival because *An. gambiae* mosquitoes are adapted to humid environment (21, 65). Although mosquitoes in the highland deforested area exhibited a reduced survivorship, they showed a significantly higher net reproductive rate and per-capita intrinsic growth rate than those in the forest area. This may primarily be due to the higher fecundity and shorter generation time exhibited by mosquitoes in the deforested area. A combination of sugar and blood feeding in mosquitoes seems to affect their longevity (66).

The results presented here have implications for understanding the effect of land cover changes on malaria transmission in African highlands. Earlier studies found that deforestation can increase larval productivity, accelerate larval development time, and increase larval pupation rate as well as adult emergence rate (40). In this study, it was found that adult survival time was shorter in the deforested area than in the forest area but the net reproductive rate was greater in the deforested area than that in the forest area. Putting them all together, it was found that deforestation can, first, greatly reduce vector generation time, thus, there will be more generations of vector in this area; second, increase reproductive rate together with increased larval–pupa survival rate, thus more offspring will survive to produce even more larvae; more generations and more offspring will greatly enhance the vector population. Furthermore, increased temperatures as a result of deforestation can shorten the gonotrophic cycle length, increase biting rates, and fasten the sporogonic development of the *Plasmodium* parasite in mosquitoes (64). In terms of malaria transmission, this could result in a great increase in the vectorial capacity and, consequently, of malaria transmission.

## Conclusion

This study concludes that, the land cover changes and deforestation are the main drivers for the temperature rise in African highlands and escalates the rate of potential malaria vectors *An. gambiae* ssp., *An. funestus*, and *An. arabiensis* colonizing the highlands. It is also enhancing significantly sporogony development rate and adult vector survivorship and therefore malaria transmission risk in the highlands.

## Author Contributions

EJK and SM conceived and designed the experiments, performed the experiments, and analyzed the data. EJK, SM, and EEK wrote the paper and also read and approved the final manuscript.

## Conflict of Interest Statement

The authors declare that the research was conducted in the absence of any commercial relationships that could be construed as a potential conflict of interest.
